# Comparative evaluation of the success of Er,Cr:YSGG laser, ferric sulfate, or a herbal hemostatic agent for hemostasis in primary molar pulpotomy

**DOI:** 10.1111/eos.70022

**Published:** 2025-06-05

**Authors:** Necibe Damla Şahin, Volkan Arikan, Firdevs Tulga Öz

**Affiliations:** ^1^ Faculty of Dentistry Department of Pediatric Dentistry Tokat Gaziosmanpaşa University Tokat Turkey; ^2^ Faculty of Dentistry Department of Pediatric Dentistry University of Kırıkkale Kırıkkale Turkey; ^3^ Faculty of Dentistry Department of Pediatric Dentistry University of Ankara Ankara Turkey

**Keywords:** blood coagulation, laser therapy, plant extracts, primary teeth, pulp tissue

## Abstract

The objective of this study was to evaluate the clinical and radiographic success of the use of the Er,Cr:YSGG laser, ferric sulfate, or a herbal hemostatic agent for hemostasis in primary molar pulpotomy. Sixty‐five children aged 5–9 years with a total of 81 primary mandibular second molars indicated for pulpotomy were included in the study. The teeth were randomly divided into the following three groups according to the pulpotomy agents used: ferric sulfate, herbal hemostatic agent, or Er,Cr:YSGG laser. Following pulpotomy treatments, the patients were followed up for 12 months. Pearson Chi‐square and Fisher's exact tests were used for statistical analysis. At the end of the follow‐up period, the clinical success rates for ferric sulfate, the herbal hemostatic agent, and the Er,Cr:YSGG laser were 96, 100, and 100%, respectively, while the radiographic success rates were 76, 73, and 96%, respectively. There was a statistically significant difference between treatments with respect to radiographic success rates, with the Er,Cr:YSGG laser showing a higher success rate compared with the other groups. Based on the results of the present study, it can be concluded that the Er,Cr:YSGG laser can be used as a hemostatic agent in the pulpotomy of primary teeth and is considered a suitable alternative to ferric sulfate and the herbal hemostatic agent.

## INTRODUCTION

Pulpotomy may be the treatment of choice for deciduous teeth with extensive caries involving the dental pulp before the expected time of exfoliation. Pulpotomy is a treatment protocol aiming to maintain residual radicular pulp vitality after the complete removal of the coronal pulp tissue [1]. During the pulpotomy treatment, each of the procedural steps, such as isolation, complete removal of the coronal pulp tissue, achievement of hemostasis, capping of canal orifices with a suitable material and final restoration are important factors in terms of affecting the success of the pulpotomy treatment [2]. Among these factors, the achievement of hemostasis is one of the most critical steps, because the residual blood clot and hemorrhage have been reported to prevent pulpal healing and to initiate an inflammatory response through the release of chemotactic factors [3, 4]. For this reason, the use of hemostatic agents to achieve pulpal bleeding control with minimal clot formation has been reported to increase the treatment success during hemorrhage control [5, 6]. Accordingly, the use of hemostatic agents has gained popularity in recent years [6].

Ferric sulfate, widely used in the pulpotomy treatment of primary teeth, provides hemostasis through a chemical reaction with blood [7]. The metal–protein complex, formed due to contact with blood, creates a mechanical plug; thus, hemostasis occurs without clot formation [8].

The Ankaferd Blood Stopper (Ankaferd), recently introduced to clinical practice as an alternative to ferric sulfate, is a herbal hemostatic agent. It consists of a standardized mixture of *Thymus vulgaris* (thyme), *Alpinia officinarum* (galangal), *Vitis vinifera* (vine), *Glycyrrhiza glabra* (licorice), and *Urtica dioica* (nettle). Each of these plants affects blood cells, endothelium, angiogenesis, vascular dynamics, cellular proliferation, and cell mediators. The herbal hemostatic agent manifests its hemostatic effect by blood proteins and erythrocytes, primarily fibrinogen, forming a “protein network” within the plasma and serum [9]. This process contributes to clot formation and the formation of a plug, effectively stopping the bleeding.

Another alternative technique introduced to clinical practice in recent years to provide hemostasis in vital pulp treatment is the use of lasers with various wavelengths. Using lasers in pulpotomy provides many advantages such as tissue vaporization and coagulation, minimal clot formation, maintenance of a blood‐free working field due to the ability of the laser to close small blood vessels, and sterilization of the wound surface. Furthermore, laser systems have been reported to induce cellular biostimulation [10, 11]. These advantages of lasers may contribute directly to the clinical success of pulpotomy procedures. The combination of these effects has the potential to accelerate the healing process, reduce the risk of postoperative complications, and to enhance long‐term treatment outcomes [12]. Er,Cr:YSGG lasers, which belong to the Erbium laser family, are the laser systems with the highest absorption rate by water, having a wavelength of 2780 nm. Besides, Erbium lasers manifest bactericidal effects [12, 13].

Although the use of hemostatic agents improves the success of primary tooth pulpotomy, studies comparing these agents are lacking in the literature. There is also a lack of data on the success of the Er,Cr:YSGG laser. The aim of this study was to evaluate the clinical and radiographic success of the use of the Er,Cr:YSGG laser, ferric sulfate, and a recently popular herbal hemostatic agent for hemostasis in primary molar pulpotomies.

The null hypothesis of the study was that there is no statistically significant difference in the clinical and radiographic success rates when Er,Cr:YSGG laser, ferric sulfate, and herbal hemostatic agent are used for hemostasis in primary molar pulpotomy.

## MATERIAL AND METHODS

### Study design

The study was designed as a three‐arm parallel randomized controlled clinical trial of three methods for hemostasis in primary molar pulpotomy with a 1‐year follow‐up period. The trial was conducted at the Department of Pediatric Dentistry of Kırıkkale University, between April 2018 and October 2019.

### Ethical approval

Approval for the study was obtained from the Kırıkkale University Faculty of Medicine Clinical Research Ethics Committee (Decision # 08/10–date: April 03, 2018). A retrospective clinical trial registration was performed at https://clinicaltrials.gov (NCT06403306).

Before the study, all eligible patients and their parents were informed about the treatment and the study, and they signed the Informed Voluntary Consent Form following their approval and consent to participate. The research was conducted in full accordance with ethical principles including the World Medical Association Declaration of Helsinki.

### Sample size and power calculation

The sample size calculation was performed using g*power (ver. 3.1.9.7) software (https://www.psychologie.hhu.de/arbeitsgruppen/allgemeine‐psychologie‐und‐arbeitspsychologie/gpower). An effect size of *w* = 0.393, a Type‐I error rate (*α*) of 0.05, and a power (1 − *β*) of 0.80 were assumed. Accordingly, the minimum required sample size was determined to be 63 primary second molar teeth [14]. However, to account for potential losses during follow‐up, the study was planned to include 81 teeth.

### Study population and inclusion criteria

The study was carried out on 65 patients (33 females and 32 males) with ages ranging between 5 and 9 years and having no systemic disease. The patients were included in the study after careful clinical and radiographic examination for the following inclusion criteria: (i) presence of a deep dentin caries lesion in a primary mandibular molar extending to the inner quarter of the dentin with a high likelihood of pulp exposure during caries removal; (ii) signs and symptoms of healthy pulp or reversible pulpitis (e.g., lack of spontaneous pain; lack of prolonged pain following thermal stimuli; healthy lamina dura and periodontal space; no radiographic pathologies in the interradicular and periapical regions; no pathological external and internal resorption; no sensitivity to percussion and palpation; no soft tissue pathologies such as edema, fistula, and abscess); (iii) suitability for restoration with stainless steel crowns, and no calcified masses within the pulp [1, 15, 16]. Only the mandibular primary molars were considered eligible for the study to evaluate the roots better radiologically. The same postgraduate student obtained the medical history and performed the examinations of all potentially eligible children, and an experienced clinical faculty member verified the diagnosis. The researchers were calibrated by evaluating a randomly chosen 10 patients in two different sessions regarding inclusion criteria (kappa value = 0.9).

The 65 patients harbored a total of 81 mandibular primary molars indicated for pulpotomy, and these teeth were randomly divided into three groups according to the indicated hemostatic agent, ferric sulfate (*n* = 27), the herbal hemostatic agent (*n* = 27), and a Er,Cr:YSGG laser (*n* = 27). The randomization procedure was performed using https://www.randomizer.org, and the additional teeth were included in the study to safeguard against possible losses throughout the follow‐up period. In the randomization list that was prepared before the clinical procedures, each tooth was assigned a number, and the group allocation list was prepared accordingly. The operator, who also performed the treatments and follow‐up evaluations, prepared and strictly followed the randomization schedule and each allocation was confirmed by an experienced clinical faculty member to ensure that the allocation sequence was strictly adhered to, and no errors were made during the assignment process.

### Treatment procedure

A single physician performed all the patients' treatments. Following the administration of local anesthesia, the teeth were isolated by a rubber dam. The carious enamel tissue was removed, and the cavities were formed using a high‐speed rotary instrument (KaVo) under water cooling. The carious dentin was removed using a low‐speed rotary instrument (KaVo) and a steel round bur (Hager and Meisinger). In teeth with a pulp exposure greater than pinpoint or carious pulp exposure, an access cavity was opened; the coronal pulp was removed with a sharp, sterile excavator and low‐speed rotary instrument; and the pulp chamber was rinsed with saline. Primary bleeding control was provided in all groups by applying moisturized sterile cotton pellets over the canal orifices for 5 min with minimal pressure. Teeth in which bleeding could not be controlled within 5 min were excluded from the study, and root canal treatment was performed in these teeth [1]. In the teeth where bleeding control was provided and the pulp was judged to be vital without suppuration, purulence, and necrosis, ferric sulfate, the herbal hemostatic agent, or the Er,Cr:YSGG laser was applied according to the order on the randomization list as described as follows. After the primary bleeding control, the ferric sulfate solution (Viskostat; Ultradent,) was kept in the cavity for 15 s using cotton pellets. The herbal hemostatic solution (Ankaferd Blood Stopper, Ankaferd) was drawn from the 1 mL ampoule to the syringe according to the manufacturer's instructions and was kept in the cavity for 15 s using cotton pellets. In the laser group, both the physician and the patient wore protective glasses before the procedure. The Er,Cr:YSGG laser (Waterlase MD, Biolase) was applied in a noncontact manner on the pulp tissue from a distance of 3–4 mm for 10 s, with an energy intensity of 25 mJ, output power of 0.5 W, and frequency of 20 Hz [10]. A MZ6 laser tip (the fiber tip 600 mm in diameter) was used for application. The laser tip was replaced with a new one following every five procedures to avoid deformation of the sensitive MZ6 fiber tip that would affect the study results. In all groups, after this secondary bleeding control, the pulp chamber was sealed with zinc oxide eugenol (Cavex). Glass ionomer cement (Ionofil U, Voco) was applied over the zinc oxide eugenol base and teeth were restored with stainless steel crowns (3 M ESPE).

### Follow‐up

Following the treatment, the patients were re‐called for follow‐up examinations at intervals of 3 months for a total of 12 months. Each patient was evaluated regarding clinical and radiologic treatment success by two different calibrated researchers independently. Treatment was considered clinically unsuccessful when one of the following findings were made: spontaneous pain, tenderness to percussion and palpation, fistula formation, soft tissue swelling, or pathological mobility. In the radiologic examination, the presence of periapical and/or interradicular radiolucency, widening of the periodontal ligament, loss of lamina dura, the presence of internal or external pathological resorption were considered to indicate failure. In teeth in which treatment was considered unsuccessful, root canal treatment or extraction was performed according to the cause of the failure. When indicated, a space maintainer was applied following extraction.

### Statistical analysis

Statistical analysis of the data was performed using the spss 23.0 package program (IBM). The data were analyzed at various follow‐up periods. Pearson's Chi‐square test and Fisher's exact test were used to evaluate periodic clinical and radiographic success rates. The level of statistical significance was set at *p* < 0.05.

## RESULTS

**FIGURE 1:**
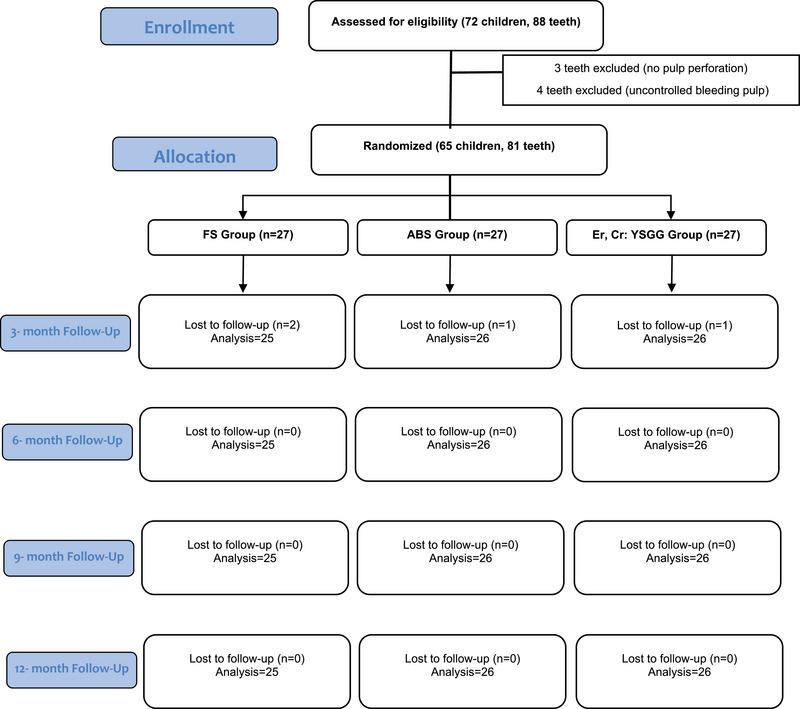
CONSORT diagram showing the flow of patients and teeth treated by pulpotomy up to 12‐months follow‐up.

This study was initiated with a total of 88 teeth in 72 patients, which were diagnosed with deep dentin caries that were suggested to require pulpotomy treatment as a result of the clinical and radiographic evaluation. Following caries removal, three primary molars were excluded because no pulp exposure had occurred. In addition, four primary molar teeth were excluded from the study due to uncontrollable bleeding at the canal orifices, and canal treatment was performed on those teeth. Thus, a vital pulpotomy treatment was performed in 81 mandibular primary second molars of 65 patients (33 females and 32 males). Three patients (four teeth) were lost to follow‐up as they were not present for their follow‐up examination. As a result, the study was conducted with 77 teeth of 62 patients (32 females and 30 males) consisting of the ferric sulfate group (*n* = 25), the herbal hemostatic agent group (*n* = 26), and the laser group (*n* = 26). Table 1 shows the distribution of pulpotomy agents according to gender, age, and tooth type, while Figure 1 presents the flow chart of teeth treated with pulpotomy.

**TABLE 1:** Distribution of pulpotomy agents by age, gender, and tooth type.

*n* (%)	Ferric sulfate	Herbal hemostatic agent	Er,Cr:YSGG laser	Total
Age (mean)	6.44	6.81	6.50	6.58
Gender				
Male	14 (56%)	14 (54%)	14 (54%)	42 (55%)
Female	11 (44%)	12 (46%)	12 (46%)	35 (45%)
Type of tooth				
Left second molar	12 (48%)	14 (54%)	16 (62%)	42 (55%)
Right second molar	13 (52%)	12 (46%)	10 (38%)	35 (45%)

At the end of the 12‐month follow‐up period, the clinical success rate was 96% in the ferric sulfate group and 100% in the herbal hemostatic agent and laser groups. No statistically significant difference was determined among the groups regarding the clinical success rates (*p* = 0.325).

**FIGURE 2:**
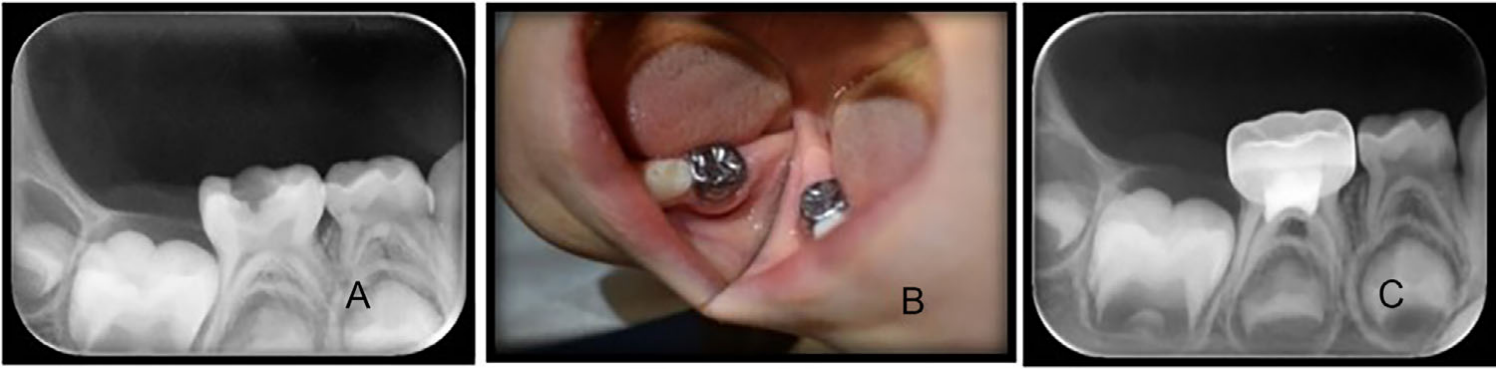
Images showing the right mandibular second molar that failed both clinically and radiographically in the ferric sulfate group. (A) Preoperative radiograph. (B) Intraoral abscess at the 3‐month follow‐up. (C) Interradicular lesion at the 3‐month follow‐up.

One tooth in the ferric sulfate group, which presented with an abscess and an interradicular lesion at the 3‐month follow‐up, was classified as clinically and radiographically unsuccessful (Figure 2).

**FIGURE 3:**
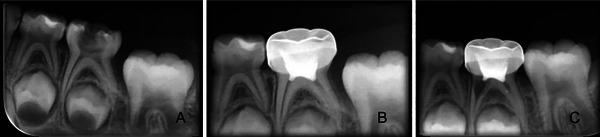
Images of the internal resorption on the left mandibular second molar in the herbal hemostatic agent group. (A) Preoperative radiograph. (B) 3‐month follow‐up. (C) 6‐month follow‐up.

**FIGURE 4:**
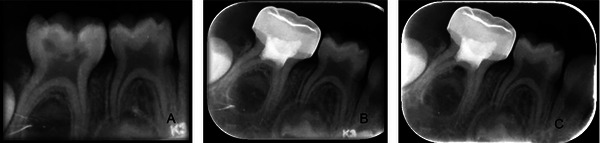
Images of the radiographic failure in the Er,Cr:YSGG laser group. (A) Pre‐operative radiograph. (B) 3‐month follow‐up. (C) Interradicular lesion at the 6‐month follow‐up.

At the end of the 12‐month follow‐up period, the radiographic success rate was determined as 76% (19/25) in the ferric sulfate group, 73% (19/26) in the herbal hemostatic agent group, and 96% (25/26) in the laser group. At the end of the 12‐month follow‐up period, comparisons between the two treatments revealed that the laser had a statistically significantly higher radiographic success rate compared with both ferric sulfate (*p* = 0.037) and the herbal hemostatic agent (*p* = 0.021). However, there was no statistically significant difference between the ferric sulfate and the herbal hemostatic agent (*p* > 0.811). A tooth with internal resorption in the herbal hemostatic agent group is shown in Figure 3, and a tooth with an interradicular lesion in the laser group is shown in Figure 4. The causes of failure encountered throughout the follow‐up period are presented in Table 2.

**TABLE 2:** Number of radiographic failures of pulpotomy agents during follow‐up periods.

Pulpotomy method	Follow‐up period (months)	Causes of failure	
		Internal resorption	Interradicular lesion
Ferric sulfate (*n* = 25)	3	1	3
	6	1	1
	9	–	–
	12	–	–
Herbal hemostatic agent (*n* = 26)	3	3	–
	6	2	1
	9	1	–
	12	–	–
Er,Cr:YSGG laser (*n* = 26)	3	–	–
	6	–	1
	9	–	–
	12	–	–

## DISCUSSION

The control of pulpal bleeding is one of the crucial steps during pulpotomy treatment. When bleeding is not controlled, the blood clot forming on the pulpal surface might create a barrier between the pulp tissue and the sealing material, resulting in a chronic inflammatory response. Besides, the space forming between the sealing agent and pulp tissue may lead to leakage, causing secondary infection [17]. The most widely used bleeding control method is to apply mechanical pressure with a moisturized cotton pellet on the pulp tissue [18]. The use of hemostatic agents during hemorrhage control to prevent clot formation, which is a disadvantage of these conventional methods, has recently gained interest [2, 19, 20].

The aim of this study was comparatively to evaluate the clinical and radiographic success rates using Er,Cr:YSGG laser, ferric sulfate, and herbal hemostatic agent, all of which are thought to minimize clot formation in primary teeth pulpotomy treatments. At the end of the 12‐month follow‐up period, the laser group showed a statistically significantly higher radiographic success rate compared with the other two groups. Therefore, the null hypothesis of the study was rejected.

In our study, while the clinical success rates of the ferric sulfate, the herbal hemostatic agent, and Er,Cr:YSGG laser groups were 96, 100, and 100%, respectively; the radiographic success rates were 76, 73, and 96%, respectively. Statistical analysis showed no significant difference in clinical success among the groups, whereas the laser group demonstrated a significantly higher radiographic success rate compared with the other two groups. These results are consistent with previous opinions that radiographic pathologies might not have been revealed immediately [21, 22], and thus, the clinical success rates might have been higher than the radiographic success rates [23].

Ferric sulfate, providing hemostasis by a chemical reaction with blood, has become a widely used hemostatic agent in primary molar pulpotomy treatment [7]. In line with our results, the success rates of ferric sulfate pulpotomy have been reported to be between 85 and 100% clinically [19, 24, 25] and between 43 and 100% radiographically [24, 25, 26, 27]. The different success rates in some previous studies may be the result of different methodologies, such as a different selection of final restorations and the inclusion of first primary molars along with second primary molar teeth.

The herbal hemostatic agent, which was recently introduced as an alternative to ferric sulfate, is a product based on various plant extracts. Although the number of studies with the herbal hemostatic agent is limited, overall success rates are between 85 and 95% [28, 29, 30]. Our results corroborate previous success rates which are comparable to ferric sulfate and support the conclusion that the herbal hemostatic agent can be used as an alternative to ferric sulfate in the pulpotomy of primary teeth.

In the present study, statistically significant differences were found in the clinical and radiographic success rates at the end of the follow‐up period, with the Er,Cr:YSGG laser group demonstrating a higher success rate compared with the other two groups. This result might have been related to the characteristics of Er,Cr:YSGG laser systems [31]. Er,Cr:YSGG lasers are recommended systems for hard and soft tissue procedures, provision of hemostasis and clotting [32]. Biostimulation, one of the major advantages of the Er,Cr:YSGG laser, is a common effect observed with all lasers [4, 31]. Various clinical and histological studies investigating the effects of low‐level lasers when applied to dental tissues with the purpose of biostimulation have reported that this application had effects such as increasing dentinogenesis, reducing pulpal inflammation, preserving pulpal vitality, and improving healing [33, 34]. Another significant advantage of the Er,Cr:YSGG laser is decontamination [4, 35]. Lasers manifest their antibacterial effects through the heat generated during irradiation [35]. Even so, researchers have reported that the increase in temperature does not reach a level that would damage the dental tissues [36]. Toomarian *et al*. [37] evaluated the histological results of ferric sulfate and Er,Cr:YSGG laser in an amputation study in dogs, and found that the integrity of the odontoblastic layer is better preserved with the Er,Cr:YSGG laser, and that the incidence of internal resorption, necrosis, and abscess formation is lower.

A follow‐up period of 12 months is at the lower margin for pulpotomy studies and can be considered as a limitation in the present study, and longer follow‐up periods may have resulted in significant differences between the groups. On the other hand, we found that most failures occurred within the first 6 months, and none in the last 3 months of the study.

As previously reported by many researchers [23, 27], the most common cause of failure was internal resorption in the present study, observed in eight (57%) of the 14 teeth that were considered radiographically unsuccessful. The early internal resorption observed in the ferric sulfate and the herbal hemostatic agent groups in the present study can be a result of the misdiagnosis of the pulp's pathological status, thus originating from an infection existing before the treatment was initiated. The Er,Cr:YSGG laser might have increased cellular proliferation due to its biostimulatory effect and might have suppressed the existing inflammation. We think that these positive effects might have caused the absence of internal resorption in this group.

The findings of the present study are consistent with previous research suggesting that laser hemostasis during pulpotomy may replace conventional techniques. The study's findings support the consideration of the Er,Cr:YSGG laser as a promising option for hemostasis. Er,Cr;YSGG laser systems have advantages such as noncontact application, lower temperature rise and intracavity pressure during the application, minimal tissue irritation, disinfection, and biostimulation. However, considering the costs and the clinical experience required to use lasers, their usage may be somewhat limited. It was observed that all three approaches could be safely utilized to achieve hemostasis in primary tooth pulpotomy treatments. The clinical success rates of the hemostatic herbal agent were found to be acceptable for pulpotomy treatment and would seem to provide effective hemostasis.

## AUTHOR CONTRIBUTIONS

**Conceptualization**: Necibe D. Şahin and Volkan Arikan. **Formal analysis**: Necibe D. Şahin and Volkan Arıkan. **Investigation**: Necibe D. Şahin and Volkan Arıkan. **Methodology**: Necibe D. Şahin, Volkan Arikan, and Firdevs Tulga Öz. **Writing—original draft**: Necibe D. Şahin. **Writing—review and editing**: Necibe D. Şahin, Volkan Arikan, and Firdevs Tulga Öz.

## CONFLICT OF INTEREST STATEMENT

The authors declare no conflicts of interest.
